# Effect of empowering leadership on employees’ workplace loneliness: a moderated mediation model

**DOI:** 10.3389/fpsyg.2024.1387624

**Published:** 2024-06-17

**Authors:** Lingyan Hou, Wenjing Cai

**Affiliations:** ^1^School of Public Affairs, University of Science and Technology of China, Hefei, China; ^2^Department of Management & Organization, Vrije Universiteit Amsterdam, Amsterdam, Netherlands

**Keywords:** empowering leadership, workplace loneliness, role breadth self-efficacy, leader–member conversational quality, self-determination theory

## Abstract

**Background:**

Workplace loneliness has become a prevalent experience among employees in organizations; however, there is limited empirical research on how leaders can address and mitigate this issue. Drawing upon self-determination theory and empowering leadership theory, this study examines the impact of empowering leadership on workplace loneliness by exploring the mediator of role breadth self-efficacy and the moderator of leader–member conversational quality.

**Methods:**

A time-lagged research design was used, collecting data through a two-wave online survey involving 531 employees in Chinese public sectors. The participants consisted of 321 males and 210 females, with an average age of 35 years (SD = 7.36).

**Results:**

Our findings indicate that empowering leadership positively influences employees’ role breadth self-efficacy, reducing their workplace loneliness. Moreover, leader–member conversational quality strengthens this indirect effect, suggesting that empowering leadership is more effective in reducing workplace loneliness when leader–member conversational quality is high.

**Conclusion:**

This study expands and enriches research on the antecedents of workplace loneliness from the leadership approach, providing valuable insights for organizations to implement interventions that effectively alleviate employees’ workplace loneliness.

## 1 Introduction

Humans are social beings and have a need for social interaction and intimate relationships (Ozcelik and Barsade, 2018). When these needs are not adequately met, individuals may experience loneliness (Lam and Lau, 2012). Workplace loneliness is defined as “an individual’s subjective assessment of whether their need to belong can be satisfied by the organizations and their colleagues” (Wright and Strongman, 2006, p. 59). With the rise of remote work in the post-pandemic era, workplace loneliness has become prevalent in organizations (Becker et al., 2022). Studies have shown that workplace loneliness is linked to a decline in employees’ organizational commitment (Jung et al., 2021), job performance (Ozcelik and Barsade, 2018), and creativity (Peng et al., 2017), highlighting the importance of decreasing employees’ loneliness in the workplace (Wright and Silard, 2021).

Due to the negative and widespread effects of workplace loneliness (Firoz and Chaudhary, 2022), scholars have endeavored to identify the antecedents of workplace loneliness and suggested the leadership approach by investigating the specific leadership style as an important predictor of employees’ workplace loneliness (Firoz et al., 2021). Leaders play a pivotal role in shaping employees’ mental health (Kim and Beehr, 2023), and research has consistently shown that positive leaders who prioritize employee growth and provide resources can reduce employees’ negative emotional experiences (Chen et al., 2021; Wright and Silard, 2021). Currently, existing research has examined the influence of paternalistic leaders and transformational leaders on workplace loneliness from a work-family perspective and a reciprocity perspective, respectively (Oge et al., 2018; Kloutsiniotis et al., 2022). However, these studies failed to thoroughly explain the psychological mechanisms by which leadership behaviors influence employees’ loneliness (Firoz et al., 2021), especially ignoring the role of employees’ internal motivation in reducing negative emotions (Parker, 2000). This is an important limitation requiring urgent investigations because recent research has demonstrated that individual internal resources can effectively alleviate loneliness (Anand and Mishra, 2021). Therefore, we are encouraged to further explore the intervention mechanisms of workplace loneliness from the leadership approach.

Aiming at addressing the research gaps above, we referred to the literature on workplace loneliness, and found that motivation, as a significant factor influencing employees’ mental health in the workplace, could be an essential psychological mechanism to explain how organizations (i.e., leadership behaviors) influence employees’ workplace loneliness (Parker, 1998; Gagné and Deci, 2005; Wright and Silard, 2021). Moreover, previous research found that empowering leadership was a significant contextual factor in driving individuals’ internal motivation (Audenaert and Decramer, 2018). In light of these findings, we attempt to examine the effect of empowering leadership on workplace loneliness, with the aim of identifying a more direct leadership strategy that contributes to a comprehensive understanding of the causes and prevention mechanisms of workplace loneliness.

Empowering leadership, characterized by power-sharing and autonomy, differs fundamentally from other leadership styles (Kim et al., 2018). Based on the empowering leadership theory, empowering leaders always grant employees full autonomy and advocate sharing power (Ryan et al., 2010). These empowering behaviors enable employees to feel recognized by their leaders and enhance their willingness to actively participate in workplace interactions (Kim et al., 2018), ultimately helping to reduce their workplace loneliness (Cheong et al., 2019; Arshad et al., 2022). Therefore, we argue that empowering leadership is an effective supervisory approach to reduce employees’ workplace loneliness.

We draw on self-determination theory to explore the intervening mechanism through which empowering leadership exerts effects on loneliness. Self-determination theory suggests that the social environment where individuals work can activate and promote their internal motivation, and then can positively increase their mental health (Bakker and Van Woerkom, 2017; Deci et al., 2017). We predict that empowering leaders can enhance their employees’ role breadth self-efficacy, referring to an individual’s confidence in their ability to perform a range of tasks and activities (Parker, 2000), via building supportive and autonomous environments because these leaders can fulfill their employees’ basic psychological needs (Parker, 2000; Beltrán-Martín et al., 2017). Meanwhile, role breadth self-efficacy can reflect individuals’ internal motivation to engage in workplace interactions (Parker, 1998). Employees with high role breadth self-efficacy have abundant psychological resources to take on broader work roles, which can increase their workplace interactions and reduce their workplace loneliness (Firoz and Chaudhary, 2022; Sohail and Tahir, 2023).

**Figure 1:**
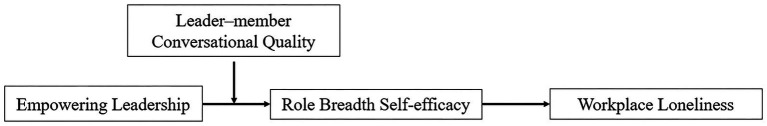
Conceptual model.

Furthermore, we suggest that leader–member conversational quality, characterized by efficiency, coordination, and accuracy of communication between leaders and subordinates (Jian et al., 2014), can strengthen the negative impact of empowering leadership on workplace loneliness. Self-determination theory points out that individual differences can significantly influence the degree to which the external social environment sparks individuals’ motivation (Deci et al., 2017). Researchers have suggested that the conversational quality perceived by employees, as a significant personal characteristic, can vary significantly across different leader-subordinate dyads (Jian and Dalisay, 2018). Specifically, we posit that in the high-level leader–member conversational quality, empowering leaders can effectively convey social cues and provide constructive feedback to their employees (Jian and Dalisay, 2018), enabling employees to successfully communicate their needs (Barry and Crant, 2000). In this condition, empowering leaders would be more effectively boost employees’ role breadth self-efficacy (Den Hartog and Belschak, 2012) by fulfilling their basic needs (Zhang and Jin, 2019), ultimately reducing their workplace loneliness (Lam and Lau, 2012). Based on the above reasoning, we propose the hypothesized model shown in Figure 1.

Our study aims to contribute to the existing literature in several ways. First, we expanded the research on the antecedents of workplace loneliness from an empowering leadership perspective and offered new leadership strategies for managers to effectively combat workplace loneliness. Second, drawing on self-determination theory, we explained the process by which empowering leaders stimulate employees’ internal motivation and highlighted the important motivational factor of role breadth self-efficacy in reducing loneliness. In this regard, we opened the black box of the leader-loneliness relationship, extending the theoretical explanation of self-determination theory in promoting self-motivation and alleviating negative emotions. Third, we provided evidence that leader–member conversational quality enhanced the negative effect of empowering leadership on workplace loneliness and confirmed that a high level of leader–member conversational quality in the workplace can strengthen the development of role breadth self-efficacy (Parker, 2000). Meanwhile, our findings emphasized the importance of the quality of the interactions between leaders and subordinates and demonstrated that the high level of conversational quality enabled leadership behaviors to exert better effects on employees’ negative emotions.

## 2 Literature review and hypothesis development

### 2.1 Workplace loneliness

Workplace loneliness is a psychological suffering at work (Wright and Silard, 2021). When employees struggle to form meaningful social and intimate connections within their organizations and are unable to cope with the absence of these relationships, they may experience workplace loneliness (Ozcelik and Barsade, 2018). With the rise of remote work and increased job competition in the post-pandemic era (Becker et al., 2022), opportunities for face-to-face and genuine socialization among employees are gradually decreasing and workplace loneliness becomes a common psychological experience in organizations (Kroll et al., 2021).

Previous studies have consistently demonstrated that workplace loneliness not only affects individuals’ attitudes and behaviors, such as decreased job satisfaction (Tabancalı, 2016), increased emotional exhaustion (Anand and Mishra, 2021), reduced creativity (Peng et al., 2017) and higher turnover rates (Chen et al., 2016), but also have a negative impact on team performance (Fu and Wen, 2021). Given its significant effects on both organizations and employees (Firoz et al., 2021), scholars have sought to identify the antecedents of workplace loneliness. Studies found that workplace loneliness was predicted by personality like core self-evaluation and shyness (Anand and Mishra, 2021; Wright and Silard, 2021), task characteristics including working hours (Heinrich and Gullone, 2006), organizational culture (Wright, 2005) and leadership behaviors (Oge et al., 2018; Kloutsiniotis et al., 2022). For instance, in organizational cultures that prioritize collaboration and compassion, employees are less likely to experience loneliness at work (Cacioppo et al., 2009).

### 2.2 Empowering leadership and workplace loneliness

Workplace loneliness is a negative emotional state that arises from inadequate relationship interactions and insufficient emotional resources (Lam and Lau, 2012; Wright and Silard, 2021). Leaders, as influential figures within organizations, have the ability to directly or indirectly influence employees’ feelings at work (Wright, 2012). Compared to authoritative leaders, empowering leaders focus on power-sharing, autonomy, and encouraging participation (Cheong et al., 2019). By granting autonomy, empowering leaders give their employees more trust, recognize the value of their contributions, and respond to their enthusiasm for work (Cao et al., 2022), which satisfies their employees’ emotional needs and helps to reduce their workplace loneliness (Zhang and Jin, 2019). Based on the empowering leadership theory, we propose that empowering leaders can better care for the needs of employees and alleviate their workplace loneliness.

Empowering leaders possess the ability to transcend hierarchical boundaries and provide employees with both job assistance and emotional care through their empowering behaviors (Biemann et al., 2015). By taking such behaviors, leaders express their appreciation and affirmation of their employees’ work (Ouyang et al., 2020), which encourages employees to develop a positive perception of their relationship with their leader and be more willing to interact positively with them, which can reduce employees’ feelings of workplace loneliness (Lam and Lau, 2012; Zhang and Jin, 2019). Furthermore, empowering leaders foster a culture of participatory decision-making, creating opportunities for their employees to actively engage in workplace interactions (Kim et al., 2018; Tang et al., 2020). Under the guidance and encouragement of empowering leaders, employees are empowered to express themselves fully and recognize that they are valued members of the organization, rather than isolated individuals, which significantly reduces their workplace loneliness (Wang and Liu, 2021; Arshad et al., 2022). Accordingly, we propose the following hypothesis:

*Hypothesis 1:* Empowering leadership is negatively related to workplace loneliness.

### 2.3 The mediating role of role breadth self-efficacy

Self-determination theory points out that the social environment where individuals work can activate and promote their internal motivation, and then can positively increase their mental health (Deci et al., 2017). Role breadth self-efficacy, defined as individuals’ confidence in taking on a wider range of job responsibilities and completing tasks beyond their assigned roles (Parker, 2000), has been found to be an important motivational factor for individuals in the work domain (Parker et al., 2006). It reflects employees’ internal motivation to engage in workplace interactions (Chiu et al., 2023) and provides positive inner resources for employees (Parker, 2000). We predict that empowering leaders can enhance their role breadth self-efficacy through building supportive and autonomous environments in the workplace, because these leaders can fulfill their employees’ basic psychological needs (Cai et al., 2018). Employees with high role breadth self-efficacy have abundant psychological resources to take on broader work roles, which can increase their workplace interactions and reduce their workplace loneliness (Lam and Lau, 2012). Accordingly, we infer that role breadth self-efficacy would serve as a mediator between the empowering leadership-workplace loneliness association.

To begin with, we predict that empowering leadership has a positive impact on employees’ role breadth self-efficacy. According to self-determination theory, individuals have three basic needs: autonomy, competence, and relatedness (Deci et al., 1994). When the external environment supports the fulfillment of these needs, it can increase employees’ internal motivation (Van den Broeck et al., 2016). We suggest that empowering leaders can better satisfy the three basic needs of employees. Specifically, empowering leaders advocate power sharing, which can better satisfy employees’ need for autonomy and enhance their sense of control within the work environment (Amundsen and Martinsen, 2014). Meanwhile, these empowering behaviors lead employees to perceive that their leaders’ trust in their ability to accomplish challenging tasks, which can boost their confidence in fulfilling their work responsibilities and meet their need for competence (Audenaert and Decramer, 2018). Additionally, through the process of empowerment, leaders can indirectly convey positive evaluations to their employees, enabling them to experience trust and respect (Arshad et al., 2022), thereby fulfilling their need for relatedness (Gagné and Deci, 2005). Consequently, empowering leaders can meet the basic needs of their employees, cultivate their confidence in undertaking diverse tasks, and increase their role breadth self-efficacy (Beltrán-Martín et al., 2017; Chiu et al., 2023).

Furthermore, we expect that role breadth self-efficacy can reduce employees’ workplace loneliness. According to the theoretical arguments of self-determination theory that internal motivation can substantially increase individuals’ mental health (Van den Broeck et al., 2016), individuals with high levels of role breadth self-efficacy demonstrate a greater willingness to undertake tasks beyond their job responsibilities, leading to a sense of fulfillment and pride in their work (Beltrán-Martín et al., 2017). In this vein, they tend to build a stronger sense of connection and belonging within the organization and ultimately reduce their workplace loneliness (Wright and Strongman, 2006). Additionally, individuals with high levels of role breadth self-efficacy possess the confidence and internal resources to actively participate in positive organizational behaviors within the workplace (Kang et al., 2022). For instance, they are more inclined to leverage their expertise and experience to assist their colleagues in task completion (Den Hartog and Belschak, 2012). These behaviors make it easier for them to develop satisfying social relationships and experience fewer feelings of loneliness (Firoz and Chaudhary, 2022).

Taken together, based on the theoretical argumentations of self-determination theory, empowering leaders can enhance their employees’ role breadth self-efficacy in supportive and autonomous environments because these leaders can fulfill their employees’ basic psychological needs (Kim and Beehr, 2023). Employees who have high levels of role breadth self-efficacy possess abundant confidence and internal resources to effectively engage in workplace interactions and thus feel less workplace loneliness (Wang and Liu, 2021). Therefore, we propose the following hypothesis:

*Hypothesis 2*: Role breadth self-efficacy mediates the relationship between empowering leadership and workplace loneliness.

### 2.4 The moderating role of leader–member conversational quality

Leader–member conversational quality refers to the efficiency, coordination, and accuracy of communication between leaders and subordinates (Jian and Dalisay, 2018), reflecting the mutual interaction quality between them (Jian et al., 2014). Self-determination theory suggests that individual differences can significantly influence the degree to which the external social environment sparks individuals’ motivation (Deci et al., 2017). Previous research has shown that the conversational quality perceived by employees, as a significant personal characteristic, can vary significantly across different leader-subordinate dyads (Jian and Dalisay, 2018). That is, when supervised by empowering leaders, employees with different conversational quality with the leaders may interpret the leaders’ message and grasp the leaders’ intentions in distinct ways (Jian et al., 2014). Therefore, we predict that leader–member conversational quality would act as a significant moderator, strengthening the relationship between empowering leadership and role breadth self-efficacy.

More specifically, in the high-level leader–member conversational quality, empowering leaders can convey social information and provide constructive feedback to their employees in a more effective way (Jian and Dalisay, 2018). Employees thus can successfully communicate their needs to their leaders (Barry and Crant, 2000). In this condition, empowering leaders would be more effectively prompt employees’ role breadth self-efficacy (Den Hartog and Belschak, 2012) via fulfilling their basic needs (Zhang and Jin, 2019). Moreover, when employees perceive a high level of leader–member conversational quality, characterized by mutual cooperation and trust (Jian et al., 2014), employees become more receptive to the information delivered by their leaders and tend to view the information provided by empowering leaders as a valuable learning opportunity to affirm themselves and improve their abilities (Cai et al., 2018). This, in turn, helps employees build confidence in their own competence and increases their role breadth self-efficacy (Kang et al., 2022).

Conversely, in the low level of leader–member conversational quality, the exchange of information between leaders and subordinates becomes inefficient and one-way, impeding the effective communication process (Jian and Dalisay, 2018). In this condition, employees may be less likely to respond positively to supportive behaviors from empowering leaders, and the relational link between leaders and subordinates weakens (Barry and Crant, 2000). Empowering leaders are difficult to fully satisfy employees’ three basic needs (Chiu et al., 2023), leading to lower levels of employees’ role breadth self-efficacy (Kang et al., 2022; Sohail and Tahir, 2023). Furthermore, when employees perceive a low level of leader–member conversational quality, they are unable to fully understand the intentions of their leaders (Jian and Dalisay, 2018), which can weaken the positive effect of empowering leaders on employees’ role breadth self-efficacy (Beltrán-Martín et al., 2017). Based on these arguments, we propose the following hypothesis:

*Hypothesis 3:* Leader–member conversational quality moderates the positive relationship between empowering leadership and role breadth self-efficacy, such that when the leader–member conversational quality is higher, this relationship becomes stronger.

### 2.5 The moderated mediation model

According to the self-determination theory, when external situational factors better satisfy employees’ three basic psychological needs, they will promote the generation of motivation, which helps them adapt to workplace changes with a positive mindset (Deci et al., 2017). Based on the previous hypotheses, it can be inferred that leader–member conversational quality moderates the indirect effect of empowering leadership on workplace loneliness through role breadth self-efficacy.

More specifically, in the high level of leader–member conversational quality, employees’ autonomy, competence, and relatedness needs can be fully satisfied by empowering leadership, and their own role breadth self-efficacy can be increased through empowering behaviors (Cheong et al., 2019). Employees with high role breadth self-efficacy can feel accepted by organizations and are more willing to increase their workplace interactions (Kang et al., 2022), thereby helping to reduce their workplace loneliness (Anand and Mishra, 2021). Conversely, in the low level of leader–member conversational quality, effective two-way communication between leaders and subordinates is lacking (Jian and Dalisay, 2018). It becomes more difficult for them to establish emotional trust with their leaders, which hinders the positive effects of empowering behaviors (Barry and Crant, 2000). In this condition, employees may struggle with negative perceptions of their competence and lack confidence in handling diverse tasks (Parker et al., 2006). When employees lack sufficient internal motivation and external incentives to actively integrate into the organization, feelings of workplace loneliness can emerge (Peng et al., 2017). Based on the above, we propose:

*Hypothesis 4*: Leader–member conversational quality positively moderates the indirect relationship between empowering leadership and workplace loneliness through role breadth self-efficacy, such that the relationship becomes stronger when the leader–member conversational quality is higher.

## 3 Methods

### 3.1 Sampling and procedure

The relevant data for our study were collected from public employees in an eastern Chinese city. To minimize common method bias caused by homogenous data, we employed a time-lagged research design by submitting two-wave questionnaires. We invited four MPA students from different public sectors in our research team to assist in the distribution of questionnaires. The four public sectors where they were working were selected by a convenience sampling method. These four public sectors include education, health, finance, and non-profit organizations. With the help of personnel department heads, we created a WeChat group with 200 employees in each of the four public sectors. All participants were aware of the role of the personnel department heads in facilitating the survey. The questionnaires were sent to public employees through an online link generated by Wenjuanxing, an online survey platform widely used in academic research in China (Chung and Meng, 2024). The study was conducted in accordance with the Declaration of Helsinki and the national regulations. We adhered to all ethical research rules required for quantitative surveys and informed consent was obtained from all individuals involved in the study. The participation was completely voluntary, and participants were informed that their responses would be anonymous and confidential, allowing them to provide honest responses based on their true feelings.

We referred to the research design of top journal articles which have successfully employed a time-lagged research design to investigate the impact of leadership behavior on employee’s psychology and organizational behaviors. These studies have consistently shown that the duration of leadership influence is approximately two months (Zhang and Zhou, 2014; Duan et al., 2017). Therefore, we followed their time-lagged research designs and set 2-month interval between time 1 and time 2. At time 1, a total of 800 questionnaires were distributed, with 200 assigned to each sector. Participants were asked to report on empowering leadership, leader–member conversational quality, and their demographic information. We received 715 valid questionnaires, a response rate of 89.38%. Subsequently, two months later at time 2, participants who had completed the first round of questionnaires were invited to report on their role breadth self-efficacy and workplace loneliness. We received 542 valid questionnaires, a response rate of 75.80%. After matching the participants’ two rounds of responses, a total of 531 valid questionnaires were received, resulting in a valid response rate of 74.3%.

Among the 531 participants, 321 were males (60.5%) and the average age of the respondents was 35 years old (*SD* = 7.36). 135 participants from the education sector (25.4%), 128 from the health sector (24.1%), 137 from the finance sector (25.8%) and 131 from a non-profit organization (24.7%). For work tenure, 5.8% (*N* = 31) had less than 1 year of work experience, 34.1% (*N* = 181) had 1–5 years of work experience, 28.8% (*N* = 153) had 6–10 years of work experience, and 31.3% (*N* = 166) had more than 10 years of work experience. In terms of education, participants included 356 undergraduate students (67%), 47 master’s students (8.9%), 4 doctoral students (0.8%), and 124 people with a high school education or below (23.4%).

### 3.2 Measures

Since the original versions of all the scales used in our study were in English, the back-translation method was adopted to accurately translate all the scales from English to Chinese (Brislin, 1970). Before the formal distribution of questionnaires, we conducted a small range of pre-tests, and according to the feedback results, we revised the ambiguous and difficult-to-understand items.

#### 3.2.1 Empowering leadership

We used a 10-item scale developed by Ahearne et al. (2005) to measure empowering leadership. The representative item was “My manager makes many decisions together with me.” Participants were asked to rate each item on a 5-point Likert scale, from 1 (strongly disagree) to 5 (strongly agree). The Cronbach’s α was 0.93.

#### 3.2.2 Role breadth self-efficacy

The 7-item scale compiled by Parker et al. (2006) was adopted to measure employees’ role breadth self-efficacy. A sample item was “How confident would you feel presenting information to a group of colleagues (1 = not at all confident, 5 = very confident).” The Cronbach’s α was 0.92.

#### 3.2.3 Leader–member conversational quality

We used the Likert-7 scale with 9 items developed by Jian et al. (2014) to measure leader–member conversational quality. Participants were asked to rate on a scale from 1 (strongly disagree) to 7 (strongly agree). The representative item was “When talking about how to get things done, the conversations between my supervisor and me usually flow nicely.” The Cronbach’s α was 0.97.

#### 3.2.4 Workplace loneliness

We used the scale developed by Wright and Strongman (2006) to measure employee’s workplace loneliness. The scale was a Likert-5 scale composed of 10 measurement items. A sample item was “I often feel alienated from my colleagues (1 = strongly disagree, 5 = strongly agree).” The Cronbach’s α was 0.97.

#### 3.2.5 Control variables

Referring to existing studies, we selected the age (years), gender (1 = male, 2 = female), tenure (1 = less than 1 year, 2 = 1–5 years, 3 = 6–10 years, 4 = more than 10 years), education (1 = high school education or below, 2 = bachelor, 3 = master, 4 = doctor), and organization type (1 = education, 2 = health, 3 = finance, 4 = non-profit organization) as control variables.

### 3.3 Analytical strategy

Due to the high homogeneity of our sample, our study was not well-suited for a multi-level research design (Chen et al., 2005; Mackenzie, 2005). The four public sectors from which we collected data were all located in the same city with the same level of government and similar staff numbers, and shared organizational characteristics. Therefore, we did not employ a multi-level research design in our study. Instead, our analytical strategy was as follows:

First, we used SPSS 26.0 to evaluate common method bias, generate descriptive statistics, conduct correlation analysis, and access reliability analysis. Amos 26.0 was adopted to test common method bias and execute confirmatory factor analysis for validating the data. Subsequently, a hierarchical regression approach was employed using SPSS 26.0 to analyze the hypotheses of the mediating and moderating effects on the theoretical model. Finally, we adopted the Bootstrap method to assess indirect effects (Preacher et al., 2007), using the PROCESS program to set the 5,000 repeated samples and evaluate the value of the confidence interval (CI).

## 4 Results

### 4.1 Common method bias

In this study, to control for common method bias in the questionnaire design, we used SPSS 26.0 to conduct Harman’s single-factor test to examine the common method bias in the collected data. The results showed that a total of four factors had eigenvalues greater than 1, the total explained variance was 70.52%, and the first factor explained 39.59% of the variance, which did not exceed the recommended value of 40% (Podsakoff and Organ, 1986). Furthermore, we added a new factor, the common method bias factor, to the original model, which explained all the items (Podsakoff et al., 2003), and constructed a five-factor model in AMOS 26.0. The results indicated that compared to the four-factor model, the changes in the fitting effect of the constructed five-factor model were not significant (*ΔRMSEA* = 0.01, *ΔCFI* = 0.02, *ΔTLI* = 0.02). Therefore, the common method bias of our study was within acceptable limits.

### 4.2 Confirmatory factor analysis

AMOS 26.0 was used to conduct a confirmatory factor analysis to test the discriminant validity among four variables, namely, empowering leadership, workplace loneliness, role breadth self-efficacy, and leader–member conversational quality. The results indicated that (see Table 1) the model fit indices for the single-factor model were χ^2^/df = 14.52, CFI = 0.47, RMSEA = 0.16, NFI = 0.45, TLI = 0.47. For the two-factor model, the indices were χ^2^/df = 5.68, CFI = 0.82, RMSEA = 0.09, NFI = 0.79, TLI = 0.81. The three-factor model showed indices of χ^2^/df = 4.82, CFI = 0.85, RMSEA = 0.09, NFI = 0.82, TLI = 0.84. The model fit indices for our proposed four-factor model were χ^2^/df = 2.57, CFI = 0.94, RMSEA = 0.05, NFI = 0.90, and TLI = 0.94. Hu and Bentler (1999) found that TLI, NFI, and CFI values exceeding 0.90 suggested a good fit, and RMSEA values lower than 0.06 indicated a relatively good data fit. Therefore, the four-factor model was significantly better than the other competing models. These results revealed a good discriminant validity among the four variables.

**Table 1:** Confirmatory factor analyses.

Model	χ^2^	df	χ^2^/df	SRMR	RMSEA	CFI	NFI	TLI
Four-factor model	2091.19	813	2.57	0.05	0.05	0.94	0.90	0.94
Three-factor model	3935.06	816	4.82	0.08	0.09	0.85	0.82	0.84
Two-factor model	7624.47	818	9.32	0.24	0.13	0.67	0.65	0.65
Single-factor model	11979.68	819	14.52	0.24	0.16	0.47	0.45	0.47

*N* = 531; ^***^p < 0.001.

Four-factor model: Empowering leadership, Role breadth self-efficacy, Workplace loneliness, Leader–member conversational quality; Three-factor model: Empowering leadership + Role breadth self-efficacy, Workplace loneliness, Leader–member conversational quality; Two-factor model: Empowering leadership + Role breadth self-efficacy + Workplace loneliness, Leader–member conversational quality; Single-factor model: Empowering leadership + Role breadth self-efficacy + Workplace loneliness + Leader–member conversational quality.

### 4.3 Descriptive statistics

We employ SPSS 26.0 to perform descriptive statistics and correlation analysis. Table 2 demonstrates the mean and standard deviation of the variables and the correlation between the variables. As displaced in Table 2, empowering leadership has a negative correlation with employee’s workplace loneliness at a low level (*r* = −0.26, *p* < 0.01), role breadth self-efficacy is negatively associated with employee’s workplace loneliness at a low level (*r* = −0.25, *p* < 0.01), and empowering leadership is positively related with role breadth self-efficacy at a medium level (*r* = 0.47, *p* < 0.01). The above results provide initial support for our theoretical model.

**Table 2:** Descriptive statistics analysis and correlation coefficients.

Model	Mean	SD	1	2	3	4	5	6	7	8	9	10	11
1.Gender	1.40	0.49	–										
2.Age	34.89	7.36	−0.14^**^	–									
3.Education	1.87	0.58	−0.03	−0.21^**^	–								
4.Tenure	2.85	0.93	−0.13^**^	0.65^**^	−0.02	–							
5.Type 1 (education)	0.25	0.44	−0.03	−0.03	0.01	−0.007	–						
6.Type 2 (health)	0.24	0.43	0.003	−0.05	0.03	0.02	−0.33^**^	–					
7.Type 3 (finance)	0.26	0.44	−0.002	−0.03	0.02	−0.07	−0.34^**^	−0.33^**^	–				
8.Empowering leadership	3.49	0.72	0.03	0.15^**^	−0.09^*^	0.07	0.05	−0.008	−0.21^**^	–			
9.Role breadth self-efficacy	3.48	0.76	−0.06	0.16^**^	−0.07	0.14^**^	−0.12^**^	−0.07	−0.13^**^	0.47^**^	–		
10.Leader–member conversational quality	4.67	1.19	0.03	0.21^**^	−0.14^**^	0.12^**^	−0.05	−0.05	−0.20^**^	0.50^**^	0.81^**^	–	
11.Workplace loneliness	2.57	0.91	−0.13^**^	−0.12^**^	−0.07	−0.53	0.02	0.02	0.16^**^	−0.26^**^	−0.25^**^	−0.34^**^	–

*N* = 531; ^**^p < 0.01; ^*^p < 0.05.

### 4.4 Hypotheses testing

In the current paper, the hypotheses are tested using hierarchical regression and bootstrap methods. We analyze the data using SPSS 26.0 and the PROCESS program. Table 3 reports our results and the detailed analysis process is as follows.

**Table 3:** Hierarchical regression results.

Variable	Role breadth self-efficacy				Workplace loneliness		
	Model 1	Model 2	Model 3	Model 4	Model 5	Model 6	Model 7
Gender	−0.07	−0.10	−0.13^***^	−0.13^***^	−0.25^***^	−0.23^**^	−0.25^***^
Age	0.006	−0.001	−0.01^*^	−0.01^**^	−0.02^*^	−0.01	−0.01
Education	−0.05	−0.03	0.04	0.04	0.05	0.03	0.03
Tenure	0.06	0.08	0.08^**^	0.08^**^	0.03	0.02	0.04
Organization type 1	−0.56^***^	−0.51^***^	−0.22^***^	−0.18	0.32^***^	0.29^***^	0.21^*^
Organization type 2	−0.50^***^	−0.42^***^	−0.16^***^	−0.13	0.32^***^	0.27^*^	0.21^*^
Organization type 3	−0.57^***^	−0.37^***^	−0.05^***^	−0.02	0.55^***^	0.44^***^	0.38^***^
Empowering leadership		0.45^***^	0.10^***^	0.10^**^		−0.26^***^	−0.19^***^
Role breadth self-efficacy							−0.16^***^
Leader–member conversational quality			0.48^***^	0.48^***^			
Empowering leadership × Leader–member conversational quality				0.05^***^			
R^2^	0.13	0.30	0.69	0.70	0.08	0.12	0.14
△R^2^	0.13	0.17	0.39	0.01	0.08	0.04	0.01
F	10.90^**^	27.77^***^	127.13^***^	114.41^***^	6.85^***^	9.17^***^	9.07^***^

*N* = 531; ^***^p < 0.001; ^*^p < 0.05; Organization type 1 = education; Organization type 2 = health; Organization type 3 = finance.

As shown in Model 6 in Table 3, after controlling for the control variables of gender, age, tenure, education, and organization type, the negative effect of empowering leadership on employees’ workplace loneliness is significant (*b* = −0.26, *p* < 0.001). This finding gives support to hypothesis 1. After adding all the control variables, it can be seen from Model 2 in Table 3 that empowering leadership has a positive effect on employee’s role breadth self-efficacy (*b* = 0.45, *p* < 0.001). Role breadth self-efficacy negatively affects employee’s workplace loneliness after controlling for empowering leadership (*b* = −0.16, *p* < 0.001; Model 7 in Table 3). Further, we adopt the Bootstrap method to test for indirect effects and set up repeated sampling 5,000 times. The results indicate that the indirect effect of empowering leadership on employee’s workplace loneliness through role breadth self-efficacy is −0.10 and the 95% confidence interval (CI: [−0.16, −0.02]) does not contain zero. Thus, the mediating effect is valid and hypothesis 2 is verified.

**Figure 2:**
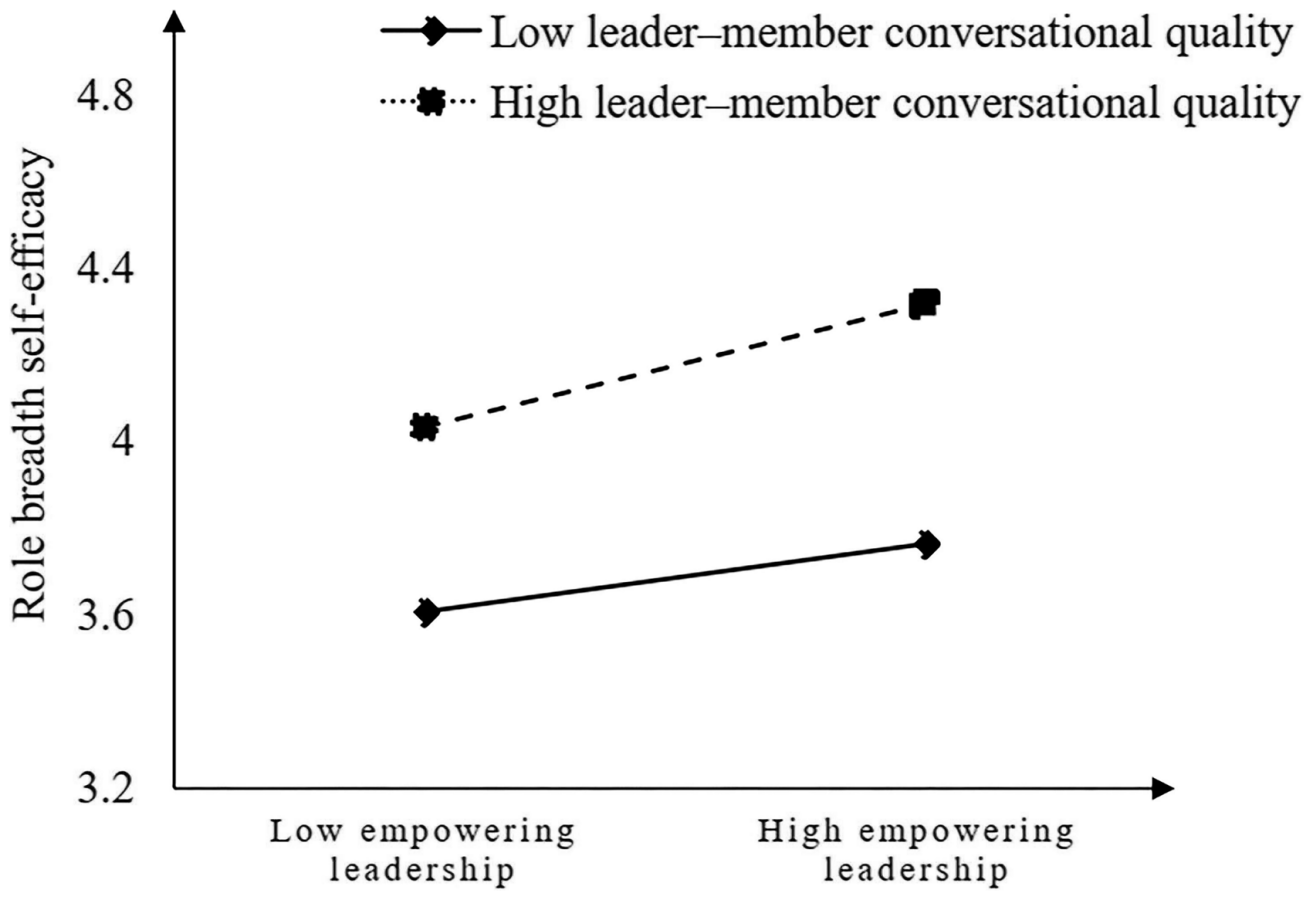
Interaction between empowering leadership and leader–member conversational quality on role breadth self-efficacy.

Hypothesis 3 proposes that leader–member conversational quality plays a moderating role in the relationship between empowering leadership and role breadth self-efficacy. As predicted, Model 4 in Table 3 specifies that the interaction term of “empowering leadership” × “leader–member conversational quality” is significant (*b* = 0.05, *p* < 0.001), which reveals that leader–member conversational quality positively moderates the relationship between empowering leadership and role breadth self-efficacy. To further explain the pattern of interactions, we also plot the simple slope diagram. The simple slope test (see Figure 2) on above (+1 SD) or below (−1 SD) 1 standard deviation indicates that when the leader–member conversational quality is high (+1 SD), the relationship between empowering leadership and role breadth self-efficacy is stronger (*b* = 0.14, *t* = 4.43, *p* < 0.001). While when the leader–member conversational quality is low (−1 SD), empowering leadership does not significantly predict role breadth self-efficacy (*b* = 0.004, *t* = 0.07, *p* >0.05). Therefore, hypothesis 3 is supported.

Further, we employ the Bootstrap method to test the moderated mediation effect and set 5,000 times of repeated sampling. We obtain the indirect effect value and 95% confidence interval of empowering leadership on workplace loneliness when the leader–member conversational quality is 1 standard deviation above and below the mean. As can be seen in Table 4, when leader–member conversational quality is low (−1 SD), the indirect effect value is −0.002 with a 95% confidence interval of [−0.02, 0.04], which contains zero, indicating that the indirect effect is not significant. When leader–member conversational quality is high (+1 SD), the indirect effect value is −0.03, with a 95% confidence interval of [−0.06, −0.005], excluding zero, indicating that the indirect effect is significant. Meanwhile, the effect value for the intergroup difference is −0.03, with a 95% confidence interval of [−0.08, −0.004], which excludes 0, making the difference significant. Thus, leader–member conversational quality significantly moderates the indirect effect of empowering leadership on employee’s workplace loneliness, and hypothesis 4 is further supported.

**Table 4:** Analysis of the moderated mediating effect.

	Indirect effect	Boot SE	95% CI
Low leader–member conversational quality (−1 SD)	−0.002	0.01	[−0.02, 0.04]
High leader–member conversational quality (+1 SD)	−0.03	0.02	[−0.06, −0.005]
Intergroup difference	−0.03	0.02	[−0.08, −0.003]

*N* = 531; Bootstrap sample size = 5,000.

## 5 Discussions

### 5.1 Overview of findings

Workplace loneliness has become a common emotional experience among employees, and organizations need to address this issue (Du et al., 2022). Drawing on self-determination theory and empowering leadership theory, this study examined the mechanisms and boundary effects of empowering leadership on employee’s workplace loneliness, considering the mediating role of role breadth self-efficacy and the moderating role of leader–member conversational quality. A time-lagged research design was used, collecting data through a two-wave online survey involving 531 public employees in China. The results indicated that empowering leadership positively influenced employees’ role breadth self-efficacy, which subsequently decreased their workplace loneliness. Moreover, leader–member conversational quality strengthened this indirect effect, suggesting that empowering leadership was more effective in reducing workplace loneliness when leader–member conversational quality was high. These findings revealed the positive influence of empowering leadership in improving employees’ negative psychological states, enriching the explanatory power of self-determination theory from the leadership approach. We also provided valuable insights for public sector managers to alleviate employee’s sense of loneliness at work.

### 5.2 Theoretical implications

First, we extended the antecedent research on workplace loneliness by examining it from the leadership approach. Existing studies have explored the correlation between leadership behavior and employee’s workplace loneliness from the perspective of paternalistic leadership and transformational leadership, respectively, (Oge et al., 2018; Kloutsiniotis et al., 2022). However, these studies failed to sufficiently indicate the psychological mechanisms through which leadership behaviors affect employees’ loneliness (Firoz et al., 2021), especially ignoring the role of employees’ internal motivation in reducing negative emotions (Firoz and Chaudhary, 2022). While recent research has demonstrated that individual internal resources can effectively alleviate loneliness (Anand and Mishra, 2021). To bridge these gaps, we found that empowering leaders can better fulfill employees’ deep-seated needs and stimulate their internal motivation to overcome workplace loneliness (Gong et al., 2009). We conducted a study with a sample of employees in public sectors using a time-lagged design to validate our findings. Our findings confirmed that empowering leadership can reduce employee’s workplace loneliness, offering a new perspective on how leadership can mitigate employees’ loneliness.

Second, drawing on self-determination theory, we empirically examined the mediating role of role breadth self-efficacy, shedding light on the mechanism that connects empowering leadership to workplace loneliness. Specifically, we explained how an externally supportive environment (e.g., empowering leadership) can stimulate individuals’ internal motivation (e.g., role breadth self-efficacy) and alleviate employees’ negative emotions (e.g., workplace loneliness). Our findings not only contributed to enhancing comprehension of self-determination theory’s effectiveness in promoting self-motivation and alleviating negative emotions, but also revealed the role that role-breadth efficacy played in adjusting employees’ psychological states.

Finally, we discovered the boundary conditions for the impact of empowering leadership on workplace loneliness. By introducing the interactive variable of leader–member conversational quality, we constructed a moderated mediation model to explore the underlying mechanisms and boundary conditions. We found that when employees perceived a high-level leader–member conversational quality, empowering leaders can more effectively meet employees’ basic needs, and prompt their role breadth self-efficacy effectively, thereby helping to reduce their workplace loneliness (Jian and Dalisay, 2018). By discussing the moderating effect, we confirmed that leader–member conversational quality strengthened the negative effects of empowering leadership on loneliness. This not only enriched our comprehension of the boundary conditions of empowering behaviors, but also substantiated the significant role of interactive contexts in mitigating employee’s workplace loneliness.

### 5.3 Practical implications

First, our findings provided valuable empirical insights for organizational managers to refine their leadership approaches, optimize human resource management, and address the issue of workplace loneliness among employees. As influential figures within organizations, leaders possess the ability to directly or indirectly influence the level of employee’s workplace loneliness. Empowering leadership, which prioritizes power-sharing and encourages active participation, enables employees to discover their own sense of value and belonging through meaningful involvement in decision-making and autonomy. Therefore, it is recommended that organizations focus on cultivating leaders’ awareness of empowerment through internal training programs. By doing so, organizations can encourage leaders to create a caring and harmonious work environment, and alleviate employees’ workplace loneliness.

Second, we recommend that organizational managers prioritized the cultivation of employees’ internal motivation and offered support in effectively addressing their psychological dilemmas. Role breadth self-efficacy, a significant psychological resource, enables employees to positively evaluate the work environment and demonstrate a willingness to devote their passion to their job responsibilities. Leaders should stimulate employees’ role breadth self-efficacy through effective management practices, ensuring they possess the sufficient emotional resources to engage in workplace interactions. Furthermore, leaders can increase communication opportunities between themselves and employees by organizing regular departmental gatherings to enhance mutual understandings and reduce their employees’ sense of loneliness.

### 5.4 Limitations and future research

First, in terms of measurement, all variables in our study were measured by self-assessment, so there was still a potential problem of data homogeneity. Future studies could use a form of data collection with multiple sources of matching, such as inviting supervisors and colleagues to conduct evaluations, to mitigate the impact of common method bias.

Second, regarding the sample, we focused on a small sample of public sectors in an eastern Chinese city. Future studies could further expand the sample size and sources to test the generalization and external validity of the findings.

Third, with regards to research design, due to the limitation of actual research resources, we adopted a time-lagged research design to explore the relationship between variables, although the research model in our paper is proposed based on theory. Future studies could consider employing experimental or longitudinal research methods to strengthen the inference of causality and make the findings more scientific.

Finally, this study has successfully identified and examined the moderating role of the leader–member conversational quality, based on self-determination theory. It is important to acknowledge that there are other factors that can also moderate the relationship between empowering leaders and workplace loneliness experienced by employees, not solely limited to leader–member conversational quality. For instance, subordinates’ personality traits, such as proactive personality and conscientiousness, may influence the effectiveness of empowering leaders (Cai et al., 2018; Jada and Mukhopadhyay, 2019). Thus, future studies could investigate the moderating role of personality traits and could reveal the boundary conditions in which empowering leaders operate from various perspectives.

## 6 Conclusion

In this study, we expand the research on the antecedents of workplace loneliness from a leadership perspective, providing insights into how empowering leaders can alleviate employees’ workplace loneliness. Drawing on self-determination theory and empowering leadership theory, we proposed a moderated mediation model that explored the relationship between empowering leadership and workplace loneliness. The results of our study indicated a negative correlation between empowering leadership and workplace loneliness, with role breadth self-efficacy as a crucial mediator in this relationship. Furthermore, our findings suggested that leader–member conversational quality strengthened this indirect effect, suggesting that empowering leadership was more effective in reducing employees’ workplace loneliness when leader–member conversational quality was high. The findings offered valuable insights into current theories and provided guidance for managers on how to alleviate employee’ loneliness.

## Data availability statement

The raw data supporting the conclusions of this article will be made available by the authors, without undue reservation.

## Ethics statement

The studies involving humans were approved by the Ethics Committee of the School of Public Affairs, University of Science and Technology of China. The studies were conducted in accordance with the local legislation and institutional requirements. The participants provided their written informed consent to participate in this study.

## Author contributions

LH: Conceptualization, Investigation, Methodology, Writing – original draft, Writing – review & editing. WC: Writing – original draft, Writing – review & editing.
